# R-Loops at Chromosome Ends: From Formation, Regulation, and Cellular Consequence

**DOI:** 10.3390/cancers15072178

**Published:** 2023-04-06

**Authors:** Yi Gong, Yie Liu

**Affiliations:** Laboratory of Genetics and Genomics, National Institute on Aging/National Institutes of Health, 251 Bayview Blvd, Baltimore, MD 21224, USA

**Keywords:** telomere maintenance, alternative lengthening of telomeres, TERRA, R-loop, RNA:DNA hybrid

## Abstract

**Simple Summary:**

R-loops, also referred to as RNA: DNA hybrids, are tripartite structures formed when a single-stranded RNA molecule forms a complex with a double-stranded DNA duplex via homology-directed base pairing. Many cellular functions are thought to be mediated by R-loops, including gene regulation, DNA replication, chromatin patterning, and DNA repair. R-loops are common and dynamic structures that account for up to 5% of mammalian genomes. In this review, we present an updated review of R-loops at a specific locus, telomeres. We summarize recent advances in the protein factors that regulate the formation and resolution of R-loops at telomeres. We also discuss the role of persistent or unscheduled R-loops in promoting genome and telomere instability and their links to human diseases.

**Abstract:**

Telomeric repeat containing RNA (TERRA) is transcribed from subtelomeric regions to telomeres. TERRA RNA can invade telomeric dsDNA and form telomeric R-loop structures. A growing body of evidence suggests that TERRA-mediated R-loops are critical players in telomere length homeostasis. Here, we will review current knowledge on the regulation of R-loop levels at telomeres. In particular, we will discuss how the central player TERRA and its binding proteins modulate R-loop levels through various mechanisms. We will further provide an overview of the consequences of TERRA-mediated persistent or unscheduled R-loops at telomeres in human ALT cancers and other organisms, with a focus on telomere length regulation after replication interference-induced damage and DNA homologous recombination-mediated repair.

## 1. Introduction

R-loops were first visualized in 1976 by electron microscopy as a three-stranded nucleic acid structure containing an RNA:DNA hybrid and a displaced single-stranded DNA [1]. This biochemical discovery was initiated with an attempt to map the 18s and 28s rRNA sequences of *Drosophila melanogaster* [2]. During the studies, Raymond and David found that, with the presence of 70% formamide in the reaction buffer and an elevated temperature, the RNA strand could invade and base-pair with one of the two DNA duplexes in the region of homology to form an R-loop. Until 1995, the in vivo significance of R-loops was demonstrated in *Escherichia coli* [3]. Two key enzymes, topoisomerase I (TOPO I) and DNA gyrase subunit B (GyrB), were found to have opposing activities on DNA supercoiling, which correlates with R-loop formation. In addition, the growth defect caused by the accumulation of R-loops can be rescued by overexpression of endoribonuclease H (RNase H). Since these discoveries, R-loops have been proven to exist in a wide range of organisms, including plants, bacteria, yeast, and mammals. R-loops are prevalent and dynamic structures that occupy up to 5% of mammalian genomes according to genome-wide profiling of R-loop sites via DNA:RNA immunoprecipitation sequencing (DRIP-seq) utilizing the S9.6 antibody (recognizing RNA:DNA hybrid) [4,5,6,7]. It is estimated that 60% of the human transcribed genome contains more than one R-loop-forming sequence according to a computational algorithm [8]. R-loops associated with transcription are mostly transient, with an estimated half-life of 10–20 min [6,9]. Using quantitative differential DNA–RNA immunoprecipitation sequencing (qDRIP-seq), a recent study extended the calculation to ~300 R-loops present in a cell at steady state, with 19 hybrids resolved every minute for a total of 27,000 per day [9].

Due to the fast resolution and short lifespan of hybrids, cells have developed multiple mechanisms to balance their formation and resolution [10,11,12,13,14,15]. Briefly, the first-layer mechanism prevents the formation and accumulation of spontaneous/transient R-loops. Earlier studies using yeast THO-complex mutants suggested that RNA processing and export factors play an important role in preventing hybrid accumulation [16,17]. Furthermore, DNA topoisomerases have been shown to be required for the resolution of DNA torsional stress caused by negative supercoiling [3,18,19]. The second-layer mechanism involves the removal of existing stable R-loops using RNA-DNA helicases and RNase enzymes. A number of helicases, including senataxin (SETX) in humans, Sen1 in yeast, DXH9, and DDX5, have been reported to directly unwind and remove RNA:DNA hybrids [20,21,22,23,24,25,26]. Among the nucleases, RNase H is the most extensively studied RNase enzyme. In eukaryotes, RNase H1 and H2 are key enzymes to remove R-loops by specifically degrading the RNA moiety of the hybrid [27]. The third-layer mechanism involves repair factors that recognize the RNA:DNA hybrids or the ssDNA in R-loops. It has been established that persistent or unscheduled R-loops often threaten DNA transcription and result in replication stress, transcription-replication conflicts, and ssDNA and dsDNA lesions in the genome [16,28,29,30,31]. As a result, various DNA repair factors and pathways are engaged to remove R-loop-mediated impairment of genome integrity. These include the nucleotide excision repair nucleases XPF-ERCC1 and XPG, which can cleave R-loops both in vivo and in vitro, as well as DNA repair proteins such as BRCA1 that recruit SETX to promote repair [32,33]. When unscheduled R-loops cause double-strand break (DSB) formation, other pathways, such as homologous recombination (HR) and non-homologous end joining (NHEJ), are employed. Lastly, a growing body of evidence suggests a mutual interplay of local chromatin state and R-loop occurrence. BRG1, the catalytic subunit of the SWI/SNF chromatin remodeling complex, acts to suppress R-loop formation by controlling chromatin accessibility [34]. BRD2 and BR4, two bromodomain proteins that recognize histone acetylation, have been shown to suppress R-loop formation via interactions with an elongation factor, pTEFb, as well as the coordinated regulation of topoisomerase activities [35,36].

Starting from their discovery, R-loops are closely associated with transcription. Based on the most widely accepted model, R-loops occur during transcription when nascent RNA “threadback” invades the DNA duplex to displace the non-template strand [37,38,39]. Recent studies have revealed that R-loop formation is closely coupled to highly expressed regions, such as rRNA and tRNA. The promoter and terminator regions of poly(A)-dependent genes are also hot spots of R-loop accumulation [5,6,20,40,41]. The function of R-loops in transcription could be stimulatory or inhibitory, and it involves multiple mechanisms ranging from direct RNA polymerase collision to indirect modifications of DNA and chromatin structure (reviewed in [10,11,42]). Aside from regulating gene expression, numerous pieces of evidence suggest that R-loops are formed and function in a variety of physiological events. For instance, R-loops are required for B cell immunoglobin (Ig) class-switch recombination (CSR), mitochondrial DNA replication, and CRISPR-Cas9 gene editing [43,44,45]. However, on the other hand, R-loops are also closely associated with human diseases. Interestingly, many diseases are associated with excessive R-loop accumulation or unscheduled R-loop formation. These include neurological or autoimmune diseases as well as cancer [12,15,46,47]. The latter is not surprising given that persistent R-loops are a source of endogenous DNA damage, which can lead to replication stress and genome instability. When they are not properly repaired, they can drive oncogenesis’s onset and progression. In the case of neurodegenerative disorders, it is believed that repeated expansion plays an important role in disease formation. For instance, R-loops are formed over expanded GAA and CGG repeats in Friedreich’s ataxia (FRDA) and fragile X syndrome (FXS) patient cells. The abundance of these R-loops not only correlates with the size of the expansion, but they also colocalize with the repressive chromatin marks that are characteristic of these diseases [48].

There have been many comprehensive and excellent reviews focused on different aspects of R-loops, ranging from mechanism to function [10,11,12,13,14,15,39,42,46,47]. Here, we present an updated review of R-loops at a specific locus, telomeres. Furthermore, we will discuss how the aberrant regulation of telomeric R-loops (or telomeric RNA:DNA hybrids) may affect telomere maintenance and its connection to human diseases.

## 2. TERRA Is the Central Player in Telomeric R-Loop Formation

Telomeres, the natural ends of chromosomes, have a central role in ensuring genome integrity by preventing the ends from being recognized as DNA breaks. Telomere dysfunction due to loss of telomeric DNA repeats or loss of protection by the binding protein complex shelterin leads to the DNA damage response, resulting in cell growth retardation, cellular senescence, or apoptosis [49,50,51]. Telomeres are organized as repetitive sequences (5′-TTAGGG-3′ in mammals) that end in a 3′ single-stranded G-rich overhang. For a long time, this terminal region was considered silent heterochromatin without gene transcription. However, in 2007, two groups reported that telomeric sequences can be transcribed, which leads to the generation of long non-coding RNAs (lncRNAs) named TElomere Repeat containing RNA (TERRA) [52,53]. Further characterization revealed that these RNA molecules are transcribed by RNA polymerase II, starting from the promoters in the subtelomeric region and proceeding from the centromere to the telomere [52,53]. Thus, TERRAs are G-rich RNA moieties containing 5′-UUAGGG-3′ repeats that are heterogeneous in size, ranging from 100 bp to more than 9 kb in mammals [52].

**Figure 1:**
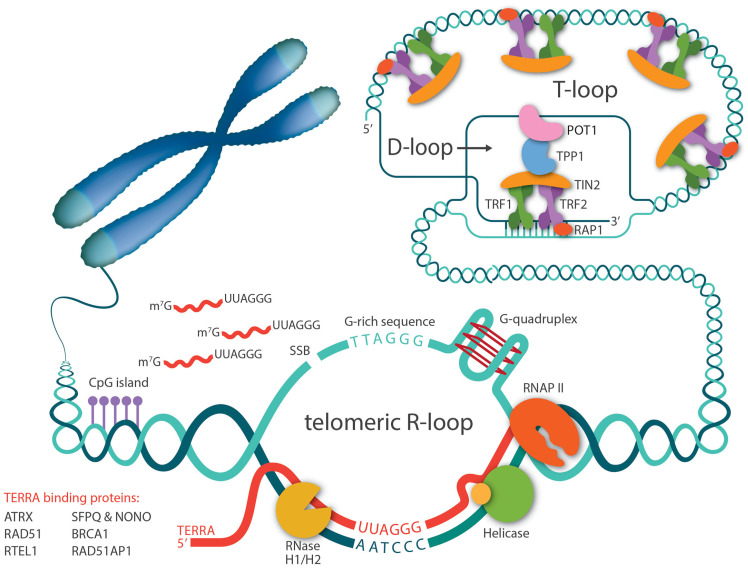
Telomeric R-loops are mediated by TERRA. TERRAs are transcribed by RNA polymerase II, starting from CpG-rich promoters in the subtelomeric region and proceeding toward telomeres. A number of proteins bind to TERRA and regulate its levels. TERRA RNA can invade telomeric dsDNA through direct base-pairing and form R-loop structures. G-rich telomeric repeats, single strand breaks (SSB), and G-quadruplex structures in telomere sequences all favor telomeric R-loop formation. General R-loop regulatory facts such as RNase H1/H2 and helicases also regulate R-loop stabilization.

Right after the discovery that the telomere has a natural transcript, TERRA, there has been speculation that TERRA can invade telomeric dsDNA and form telomeric RNA-DNA hybrids, since telomeres contain almost all the favored features for R-loop formation, including high GC skew, G-quadruplex (G4) secondary structure, and repeated sequences (Figure 1). Five years later, Lingner and Luke’s groups confirmed that R-loops occurred at telomeres in yeast and have since been detected in other organisms, including trypanosomes and mammalian cells [54,55,56,57]. In accordance with the cell cycle-dependent TERRA levels, telomeric R-loops are tightly regulated throughout the cell cycle as well [58]. Studies in *S. cerevisiae* have demonstrated that treatment with hydroxyurea, a DNA replication inhibitor, causes TERRA R-loops to accumulate in the early S phase and then decrease throughout the S phase. This decrease in R-loop levels coincides with the increase of DNA polymerase ε occupancy at telomeres, implying that TERRA R-loops are normally eliminated at the time of telomere replication to avoid transcription replication encounters.

The close relationship between TERRAs and R-loops also indicates the importance of TERRA in telomeric R-loop formation. In both yeast and mammalian cells, higher levels of TERRA correlate with increased levels of R-loops [58,59,60]. Interestingly, it was first observed in budding yeast that TERRA R-loops preferentially accumulate at critically short telomeres due to impaired degradation by RNase H and exonuclease Rat1 activity. Consequently, TERRA R-loops are stabilized and are likely to interfere with replication, which results in replication stress and DSB induction. In presenescent cells, this DDR response can further promote homology-directed repair (HDR) to elongate telomeres to prevent senescence onset [56,58]. Recently, a similar phenomenon, that TERRA R-loops preferentially associate with short telomeres, has also been reported in human cells [60]. However, the mechanism by which short telomeres recruit more TERRA R-loops remains to be identified.

It is generally accepted that endogenous R-loops are thought to occur mostly co-transcriptionally, with the RNA molecule hybridizing to its DNA template in cis. However, due to the unique long stretches of the same repetitive sequences at telomeres as well as a fraction of TERRAs presenting as the “free” nucleoplasmic form, telomeric R-loops were reported to also occur in trans in human cells [60,61]. The mechanism for this TERRA:telomeric DNA hybrid formation involves the strand exchange activity carried out by the homologous recombination protein, RAD51. Thus, unlike the early indication that R-loops are mere transcription byproducts, TERRA RNA molecules invade telomeric dsDNA independently of transcription, especially targeting short telomeres, implying that telomeric R-loops have essential functions beyond transcription.

## 3. Proteins That Regulate R-Loop Levels at Telomeres

### 3.1. General R-Loop Regulating Factors

Two proteomic searches for R-loop binding partners have identified more than hundreds of proteins [24,62]. Even though the studies were performed in different cells using different methodologies (in vivo affinity purification with the S9.6 antibody and in vitro pull-down with biotinylated synthetic hybrids), approximately 200 overlapping proteins were identified. Proteins that are shared by both approaches include RNA processing proteins, helicases, and chromatin-associated proteins. These general factors have been proven to be important in telomeric R-loop function as well. In addition to Rat1, as mentioned above, Lingner’s group reported in 2013 that all THO subunits involved in messenger RNP biogenesis and export are natural components of telomeres [55]. In budding yeast, telomeric R-loops are repressed by the THO complex. RNase H enzymes, which are known to digest the hybrid’s RNA moiety at genome-wide R-loop sites, are also involved in the removal of R-loops at yeast and mammalian telomeres [54,58]. In contrast, helicases such as Upf1, Sen1, and DDX5, which are another class of common factors, can either limit telomeric R-loop accumulation through their unwinding activity or promote hybrid structures depending on telomere length status [63].

### 3.2. TERRA Binding Proteins

#### 3.2.1. ATRX

Gibbons’ laboratory has reported that the chromatin remodeling factor ATRX negatively regulates R-loops at telomeres [64]. ATRX is part of the SWI/SNF2 family of chromatin remodeling proteins that primarily functions in the deposition of the histone variant H3.3 at heterochromatin regions such as telomeres and pericentromeric chromatin [65,66,67,68]. Though ATRX was originally discovered as the cause of the α-thalassemia, mental retardation, and X-linked (ATRX) syndrome, to date mounting evidence suggests that ATRX is mutated in a wide range of cancers, including gliomas, neuroblastomas, and sarcomas [69,70,71,72,73]. Interestingly, in most of these cancers, the loss of ATRX is intimately linked to the alternative lengthening of telomeres (ALT), a unique homologous recombination-based telomere maintenance mechanism that contributes to cellular immortality in cancer [65,74,75,76,77]. A systematic test of ATRX expression in 22 human ALT cell lines has revealed that 19 out of 22 lines have lower or complete loss of ATRX expression [78].

TERRA proteome search identified ATRX as a TERRA-associated protein, and in vitro assays later further confirmed the direct interaction [79]. The connection of ATRX to the R-loop comes with the finding that the recruitment of ATRX to telomeres is dependent on their transcription and is positively correlated with the length of the repeats. However, the abundance of TERRA R-loops is negatively correlated with ATRX recruitment. Not only does the loss of ATRX lead to increased R-loop levels, but more importantly, the reintroduction of ATRX results in a 40% reduction of RNA:DNA hybrids [64,79]. Thus, the presence of ATRX plays a critical role in suppressing R-loop formation at transcribed telomeres. Mechanistically, a recent biochemical study demonstrated that ATRX is unable to resolve R-loops in vitro under the same conditions that another helicase, DDX5, does. Instead, ATRX inhibits R-loop formation by preventing the RNA from interacting with the template DNA strand via its RNA binding activity [80].

#### 3.2.2. SFPQ and NONO

Schoeftner’s group has used a TERRA RNA pull-down approach and identified splicing factors, proline- and glutamine-rich (SFPQ), and non-POU domain-containing octamer-binding protein (NONO) proteins in mouse ES cells that maintain telomere length through both telomerase dependent and independent pathways [81]. Further investigation confirmed that both proteins co-localize with telomere repeats and are novel components of telomeric chromatin. NONO and SFPQ are proteins from the Drosophila behavior/human splicing (DBHS) family with highly conserved tandem N-terminal RNA recognition motifs (RRMs), a Nona/paraspeckle domain (NOPS), and a C-terminal coiled-coil [82]. SFPQ and NONO deletion does not change the overall levels of TERRAs (telomere-bound TERRA and free TERRA) in cells, but it does affect the telomeric intensity of TERRA foci and, more importantly, results in a significant increase of R-loops, indicating a specific role for these two proteins in TERRA homeostasis by antagonizing the formation of RNA:DNA hybrids at telomeres. Interestingly, despite the fact that SFPQ and NONO preferentially form heterodimers, these two proteins have distinct functions in suppressing R-loop-related replication stress and telomere recombination in both telomerase-positive and -negative cells. NONO is essential for preventing telomere fragility, particularly on the leading CCCTAA-repeat strand. SFPQ, on the other hand, acts as a barrier to homologous recombination at telomeres. The loss of both proteins causes massive recombination events, resulting in a 35% increase in telomere length in ALT cancer cells [81].

#### 3.2.3. RAD51

RAD51 is a well-known recombinase for facilitating strand invasion of DNA molecules during homologous recombination. It is also one of the key factors involved in the TERRA association with telomeres through the siRNA screen [60]. Notably, in vitro binding analysis revealed that RAD51 has a 3-fold higher affinity for the TERRA oligonucleotide than the corresponding telomeric DNA sequence. When RAD51 is added to the plasmid-based in vitro strand invasion assay, it can catalyze the formation of R-loops that are sensitive to RNase H1. Thus, within cells where TERRA is present at local chromosome ends, binding of RAD51 subsequently triggers the strand invasion of TERRA and promotes TERRA recruitment and R-loop formation at telomeres. The entire process is strongly analogous to strand invasion and homologous search in general DNA repair by HDR. Interestingly, using an elegant reporter construct, Lingner’s group discovered that R-loops preferentially target short telomeres in human cells, which is consistent with the observation in yeast. In addition, the authors suggested that TERRA-mediated R-loop association with telomers could occur in trans. This discovery has a significant impact on the molecular basis of telomere length homeostasis, particularly in ALT cancer cells. In human cells, TERRA transcription is detected at a subset of chromosome ends. It is estimated that 50% of TERRAs are associated with the chromosomes, with the remaining half being nucleoplasmic-free [61]. Thus, TERRA R-loop formation in trans provides an alternative mechanism for directing TERRAs to short telomeres and initiating HDR to replenish telomeric sequences in ALT cancer cells.

#### 3.2.4. BRCA1

BRCA1 is another well-known DNA repair protein that is involved in many aspects of cellular damage response, including dysfunctional telomeres [83,84,85,86,87]. It has also been implicated in general R-loop resolution [33,88,89]. The connection of BRCA1 to TERRA RNA:DNA hybrids comes from the finding that BRCA1 directly interacts with TERRA via its N-terminal nuclear localization sequence and associates with the main components of the shelterin complex in a cell-cycle dependent manner [90]. Consistent with the previous studies of BRCA1 in transcriptional regulation and DNA repair, BRCA1 is found to bind to the CpG-rich subtelomeric TERRA promoter region, suppressing TERRA transcription and R-loop formation. While at the telomeric region, BRCA1 promotes TERRA R-loop resolution likely with the help of SETX and XRN2, a 5′-to-3′ exoribonuclease known to cooperate with SETX in resolving G-rich R-loops [20,91]. Together with these two actions, BRCA1 ensures the proper abundance of TERRA R-loops at chromosome ends to suppress R-loop-mediated telomere damage.

#### 3.2.5. RTEL1

RTEL1 was discovered as a TERRA-associated protein during the proteome search that led to the discovery of ATRX. It has been reported that RTEL1 regulates R-loop resolution [79,92,93,94]. Ghisays et al. later confirmed that RTEL1 directly interacts with TERRA via its C-terminal RNA binding domain [95]. Furthermore, in vitro binding analysis revealed that RTEL1 has a strong preference (100-fold) for G-quadruplex over the mutant TERRA that is incapable of forming G-quadruplex. Notably, RTEL1 interaction with TERRA R-loops appears to be more involved in maintaining the stability of TERRA R-loops at the telomere location, unlike the proteins described earlier that influence R-loop levels through TERRA transcription or R-loop resolution. In RTEL1-deficient cells, marked elevations of TERRA levels are observed at a panel of known TERRA transcription chromosome ends. However, the telomeric TERRA signal is significantly decreased rather than increased, as assessed by TERRA RNA FISH. Furthermore, chromosome spreads reveal a significant increase in telomere loss in RTEL1 null cells. Unsurprisingly, these cells have a limited proliferative capacity and become senescent within one to two months.

#### 3.2.6. RAD51AP1

Two papers have reported that RAD51AP1 (RAD51 associated protein 1) plays an important role in promoting the R- to D-loop switch for homologous recombination and chromatin-directed mechanisms in ALT telomere synthesis [96,97]. First, an in vitro binding assay showed that purified RAD51AP1 efficiently bonds to TERRA-like oligos and can form an R-loop in the presence of a dsDNA plasmid. Moreover, RAD51AP1 exhibits 3- to 10-fold greater efficiency than RAD52 and RAD51 in forming telomeric R-loops in vitro. Yadav et al. then discovered that TERRA and RAD51AP1 contribute to telomere elongation in RAD52 knockout cells, implying that both proteins use a distinct RAD52-independent pathway for ALT telomere maintenance. Further in vitro analysis showed that RAD51AP1-catalyzed TERRA R-loop formation is essential in the initiation steps of homologous recombination in ALT cancer cells. The authors proposed that the RNA:DNA hybrid allows the displaced G-rich single strand to form G4 structures. When the RNA:DNA hybrids are removed by RNase H1 or other helicases, the resulting G4 can open telomeric dsDNA to facilitate strand invasion or D-loop formation to initiate homologous recombination-mediated telomere elongation in ALT cancer cells.

Kaminski et al. also confirmed that RAD51AP1 interacts with TERRA and proposed that RAD51AP1 is required for R-loop formation in response to DNA breaks at telomeres [97]. Using a proteomic approach, the authors found that the RAD51AP1 interactome contains multiple chromatin remodeling and modification factors. This led them to the discovery that RAD51AP1-mediated TERRA R-loops regulate chromatin by establishing transcriptionally repressive chromatin, as indicated by ubiquitination of histone H2A at lysine 119 (Ub-H2A). The significance of this reinforcement of repressive chromatin is proposed to timely stall RNA polymerase II, thereby preventing transcription-replication collisions during HDR in ALT cancer cells.

### 3.3. TERRA RNA Modification Proteins

TERRA RNA undergoes both co- and post-transcriptional modifications. As transcribed by RNA polymerase II, TERRA has a canonical 7-methylguanosine cap structure at the 5′ ends. However, the majority of TERRA (90%) lacks the poly A tail at the 3′ ends. The presence of a poly A tail largely determines the overall TERRA stability and nuclear localization. Poly(A)+ TERRA is primarily found in nucleoplasm and has low affinity for chromatin, whereas poly(A)- TERRA has a fraction (40%) associated with chromatin and the remainder (60%) in nucleoplasm [61].

TERRA, like many other eukaryotic mRNAs and non-coding RNAs, has post-transcriptional modifications at the N^6^ position of internal adenosine (m^6^A) [98]. Chen et al. showed that the methyltransferase METTL3 catalyzes this m^6^A modification, which doubles the stability of TERRAs at various chromosome ends. They also demonstrated mechanistically that modified TERRA is recognized by the m^6^A reader protein YTHDC1 for binding to maintain stability. Because depletion of either METTL3 or YTHDC1 causes R-loop reduction, HDR compromise, and telomere shortening/dysfunction, the m^6^A-modified TERRA is likely to form an R-loop with telomeres.

The RNA editing enzyme ADAR1 (Adenosine deaminase acting on RNA1) has been reported to regulate R-loop levels in non-ALT cells [99]. ADAR1 catalyzes the deamination of adenosine to inosine [100,101,102]. The ADAR1 protein exists in two forms, p150 and p110, which are generated by using two different promoters. The p150 form is mainly found in the cytoplasm and regulates interferon signaling via sensing double-stranded RNA (dsRNA). The p110 protein is mostly localized in the nucleus [103,104]. ADAR1p110 was identified as one of many R-loop binding proteins by proteomic analyses [24,62]. Nishikura’s group further characterized the role of ADAR1p110 editing in genome maintenance via R-loop formation [99]. They found that ADAR1 depletion by siRNA resulted in the accumulation of R-loops specifically at telomeres but not at α-satellite centromeric repeats or *Alu* repeats, suggesting that telomere repeats could be the major targets of ADAR1 in cells. Interestingly, ADAR1 knockdown only led to R-loop accumulation in non-ALT cancer cells, including HeLa, HEK293T, and HCT116, but not in ALT cancer cells or primary fibroblasts. Together, these findings suggest that different mechanisms of R-loop regulation may be used by ALT versus non-ALT cancer cells for continuous proliferation.

### 3.4. Shelterins

The ends of the chromosomes are shielded by a six-subunit protein complex termed shelterin [50,105]. Three subunits of the complex, TRF1, TRF2, and POT1, make direct contact with telomeric DNA sequences. The remaining three subunits, RAP1, TIN2, and TPP1, are associated with telomeres via protein-protein interactions [105]. Given that shelterin proteins play critical roles at telomeres and that deletion of TRF1 and TRF2 has been reported to alter TERRA levels [60,106,107,108], one might wonder if any shelterin component is involved in R-loop regulation. RAP1 is the first shelterin member identified as connecting to R-loops [57]. Li’s group reported that depleting TbRap1 in *Trypanosoma brucei*, a kinetoplastid parasite that causes African trypanosomiasis, results in more DSBs at telomeres and subtelomeres. They determined that this damage was caused by elevated TERRA and R-loop levels after TbRap1 removal. As a result, increased DSBs act as strong inducers of the major surface antigen, variant surface glycoprotein (VSG) switching. Notably, this type of subtelomeric plasticity is a delicate task. A limited increase in switching rate is thought to be beneficial for cell fitness, whereas severe damage at telomeres and subtelomeres is believed to be detrimental to *Trypanosoma brucei* cell viability.

In mammalian cells, TRF2 has been implicated in regulating telomeric R-loops via multiple mechanisms. In an in vitro assay, Azzalin’s group found that purified GST-TRF2 promotes TERRA-like RNA invasion and R-loop formation [109]. This is accomplished through TRF’s N-terminal basic domain, which binds to TERRA and allows for efficient TERRA invasion into telomeric dsDNA. TRF1, on the other hand, counteracts the TRF2 TERRA binding activity via its N-terminal acidic domain, implying competition or crosstalk between TRF1 and TRF2 to regulate TERRA interaction and R-loop formation at telomeres. Indeed, in both telomerase-proficient HeLa and telomerase-deficient ALT cancer cells, TRF1 depletion results in an increase in telomeric R-loops, telomere replication stress, and significant telomere loss. Lieberman’s group later observed that TRF2’s basic N-terminal Gly/Arg-rich (GAR) domain recognizes the G-quadruplex formed within TERRA [110]. Using a G4 compound that can bind to TERRA, they further demonstrated that the TERRA G4 structure is an important recognition feature for TRF2 GAR domain interaction. The G4 compound causes similar telomere shortening and fragility in cells, as described in TRF2 GAR domain deletion mutants. Lastly, TRF2 is thought to affect TERRA transcription. TERRA expression within cells is tightly regulated by the cell cycle, with levels peaking in G1 and progressively decreasing in the S phase [61]. One of the reasons is to minimize or prevent the transcription-replication conflict. Nie et al. described the underlying mechanisms and revealed that TRF2 recruits the nucleolar protein TCOF1 to telomeres during the S phase, and TCOF1 suppresses TERRA transcription and R-loop formation via binding to RNA Pol II. As expected, TCOF1 deficiency results in a significant accumulation of unscheduled R-loops and telomere replication defects. Collectively, these findings point to both positive and negative regulation mechanisms of TRF2 in keeping TERRA R-loop levels in balance within cells [111].

### 3.5. Fanconi Anemia (FA) Pathway Proteins

FA is a rare genetic syndrome characterized by progressive bone marrow failure, spontaneous chromosomal instability, and a high cancer predisposition. FA patients are hypersensitive to DNA interstrand crosslinking (ICL) agents. To date, 22 FA genes (complementation groups FANCA–FANCW) have been identified [112,113,114,115]. The protein products of these 22 genes, together with FA-associated proteins, constitute a common FA pathway for DNA ICL repair and replication fork protection under stress. Given the intricate connection between the FA pathway and replication stress, growing evidence suggests that the FA pathway is also involved in controlling R-loop accumulation and associated defects. Schwab et al. showed that RNA:DNA hybrid levels and associated damage are elevated across the genome in multiple FA mutant cells. Furthermore, the authors demonstrated that purified wild-type FANCM, one of the most conserved FA proteins, could directly resolve R-loops using its translocase activity, as evidenced by the in vitro unwinding assay, indicating the importance of FANCM enzymatic activity in resolving R-loops [116].

Three additional papers also disclosed the role of FANCM in ALT telomere maintenance by limiting the accumulation of R-loops [117,118,119]. Previous studies revealed that TERRA and R-loops are abundant in ALT cancer cells, and as a consequence, ALT cancer cells are constantly challenged by replication stress. FANCM is found to be particularly important in resolving R-loops at telomeres, as FANCM depletion results in a substantial increase in telomeric R-loop levels and telomere dysfunction-induced DNA damage signaling. All the ALT characteristic features are increased as well, including abundant extrachromosomal telomere repeat DNA and ALT-associated PML bodies (APBs). In general, deletion of FANCM is well tolerated in humans, mice, and other species [120,121,122,123]. However, in ALT cancer cells, due to the drastic increase in unscheduled R-loop accumulation and exaggerated ALT activity, cells experience rapid telomere loss and eventually stop proliferating and lose cell viability. R-loops are thus likely endogenous DNA lesions as well as physiological substrates of the FA pathway. FA proteins are required to prevent unscheduled R-loop accumulation and maintain genome integrity and telomere length.

## 4. Consequence of R-Loops at Telomeres

### 4.1. Survival Pathways in Yeast and Trypanosomes (Non-Mammalian Cells)

Budding yeast relies on telomerase to maintain telomere length, and its TERRA levels, as previously stated, fluctuate with the cell cycle [61]. Accordingly, telomeric R-loops are formed transiently and resolved rapidly to minimize the risk of transcription-replication collisions and thus have no detrimental effect on telomere integrity. Telomerase deficiency leads to progressive telomere loss during cell division and replicative senescence eventually. However, a subset of cells is able to elongate telomeres through HR-dependent recombination, thereby overcoming senescence [124]. Detailed studies further revealed that the recombination preferentially occurs at short telomeres that also display higher TERRA levels [58]. However, it is still not completely clear whether TERRA upregulation results from changes in local chromatin state, transcription, or telomeric RNase H localization, or from a combination of these mechanisms. The accumulation of TERRA leads to increased R-loop formation at critically short telomeres. If not efficiently degraded by RNase H2 or stabilized by telomere association proteins such as Npl3, stabilized TERRA R-loops are prone to induce replication stress and, as a result, initiate HR-mediated recombination for cell survival [125]. Conversely, if critically short telomeres are deprived of stabilized R-loops, either through RNase H overexpression or TERRA ablation, cells may experience an accelerated onset of senescence [55,58,125].

Trypanosomes, like yeast, use R-loop-mediated recombination to promote antigenic variation for their cell fitness. To evade the host immune responses, *Trypanosoma brucei* constantly switches its major surface antigen, VSG, to avoid elimination by the host. VSG is expressed exclusively from subtelomeric regions, where TERRA is transcribed and forms an R-loop. Recent studies have confirmed that R-loops at telomeres and subtelomeres play an important role in antigenic variation in *Trypanosoma brucei* [57,126]. In situations where TERRA/R-loop levels are increased through TbRap1 or RNase H depletion, the amount of DSBs at telomeres and subtelomeres increases accordingly, resulting in more frequent VSG switching. Elevated R-loops and DSBs are a double-edged sword. While adequate DSBs promote switching processing, excessive DSBs cause telomere dysfunction and cell death. Thus, tightly controlled R-loop levels are required to keep the balance between antigenic plasticity and telomere integrity.

### 4.2. Survival Pathways in ALT Synthesis in Neoplasia

**Figure 2:**
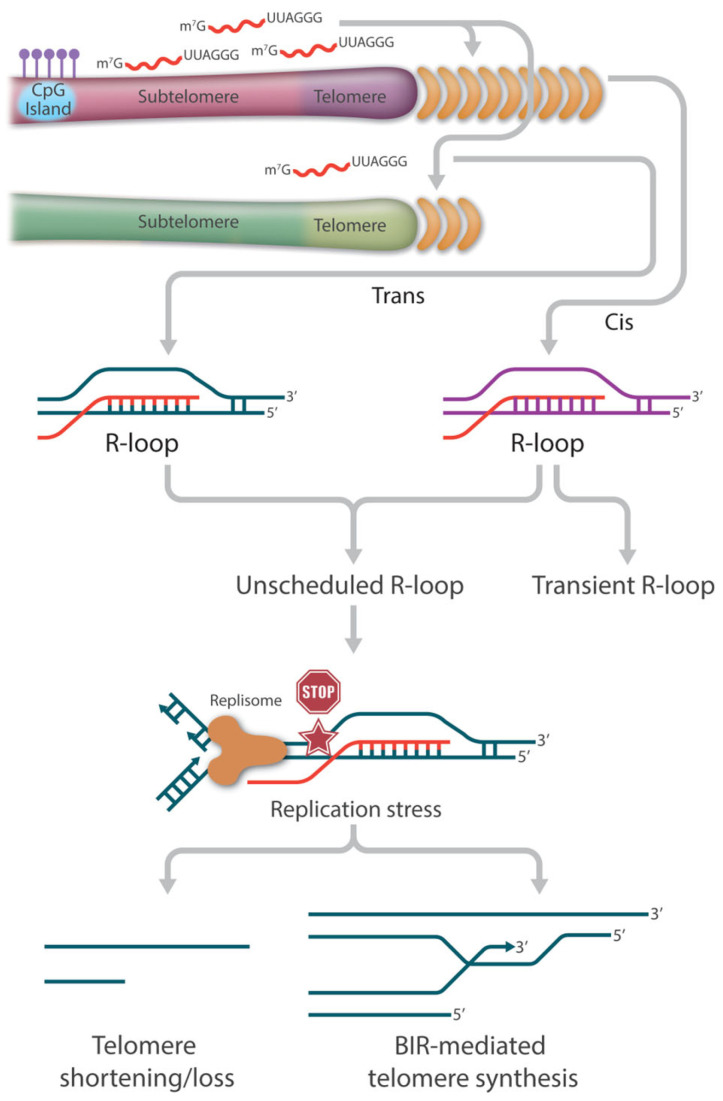
The role of TERRA-mediated R-loops in telomere length regulation. TERRA-mediated R-loop association with telomers could occur in cis and in trans. In general, telomeric R-loops are formed transiently and resolved rapidly to minimize the risk of transcription-replication collisions and thus have no detrimental effect on telomere integrity. However, the loss of balanced telomeric R-loop formation and resolution results in the accumulation of persistent or unscheduled R-loops at telomeres, which becomes one of the major drivers of replication stress. In the absence of a functional repair pathway, fork collapse and double strand breaks (DSB) can lead to telomere attrition. In the presence of a functional HR pathway, fork collapse and double strand breaks (DSB) can initiate break-induced replication (BIR)-mediated telomere synthesis.

The yeast work has led to the hypothesis that TERRA-mediated R-loops participate in similar functions to circumvent telomere shortening in telomerase-negative ALT cancer cells. It is estimated that 90% of cancers maintain their telomere length by reactivating telomerase to replenish telomere repeats at the ends of the chromosomes. The remaining 10% of cancers (or ALT cancers) can achieve replicative immortality via the HDR pathway [74,75,76,127]. Centered on the relationship between ALT and R-loops is the deregulation of TERRA. Compared to telomerase-positive cells, not only are TERRA levels higher in ALT cancer cells, but also the normal cell cycle regulation is lost [128,129,130]. The precise cause of this TERRA deregulation is not completely understood; however, it has been reported that ALT cancer cells are characterized by reduced compaction of telomeric chromatin as well as ATRX inactivation, as stated earlier [129]. Nevertheless, the ultimate increase in TERRA R-loops becomes the main source of replication stress, which is necessary for the initiation of telomere recombination (Figure 2).

Several outstanding studies have begun to unmask the dynamic features of TERRA R-loop-directed ALT synthesis. First, telomeric R-loops can be viewed as an intermediate structure in DSB processing, where TERRA can directly interact with HR proteins like RAD51 and BRCA1 to catalyze strand invasion and stimulate recombination [60]. Second, TERRA R-loops are able to initiate a RAD52-independent ALT activity by generating HR intermediates known as DR-loops, which contain both DNA:DNA and DNA:RNA hybrids [96,131]. As described earlier, Yadav et al. showed that efficient binding of RAD51AP1 to TERRA R-loops allows the formation of G4 on the displaced G-rich strand of the invaded telomeric dsDNA. Once the TERRA RNA strand gets degraded, the persistent G4 structures allow subsequent G-rich strand invasion to form telomeric D-loops. This R-to-D-loop switch facilitates HDR without the assistance of RAD52 [96]. Third, TERRA R-loops need to be removed before HDR can proceed. Although telomeric R-loops are necessary to trigger replication stress and initiate RAD51 or RAD52 strand invasion for HDR, TERRA RNA strand degradation or R-loop resolution by helicases must be coordinated in time. Finally, the HR repair proteins POLD3 and PCNA, possibly in collaboration with other proteins such as BLM, work together to complete HDR, i.e., the break-induced telomere synthesis [132,133].

### 4.3. Disease Connection of TERRA R-Loops in ICF Syndrome

ICF syndrome (Immunodeficiency, Centromeric Instability, and Facial Anomalies) is a rare human disease that is associated with genetic defects in DNA methylation [134,135]. To date, four genes, *DNMT3B* (DNA methyltransferase 3B), *ZBTB24* (zinc-finger and BTB domain-containing 24), *CDCA7* (cell division cycle associated 7), and *HELLS* (helicase, lymphoid specific), have been linked to ICF types 1–4. Patients who have typical phenotypic features of ICF syndrome without known gene mutations are referred to as ICFX [136]. Among all cases, ICF type I caused by the DNMT3B mutation is the most prevalent, accounting for almost half of all known ICF patients; it is also the most relevant for TERRA R-loops.

The majority of human TERRA promoters are located within the most distal 2kb of the genome, adjacent to the telomeric region, where the CpG percentage is disproportionately high [137]. Not surprisingly, hypomethylation caused by DNMT3B deficiency results in a drastic increase in TERRA transcription. It has been shown that total TERRA levels are 6–10 times higher in ICF cells than in healthy control cells, and they are thought to be close to 100 times higher at specific chromosome ends [138,139,140]. ChIP analysis has revealed a moderate decrease (around 5 fold) in the heterochromatin histone mark H3K9me3, but a significant increase (more than 100 fold) in the euchromatin histone modification H3K4me2, indicating a drastic change in chromatin status in the subtelomeric region [138]. ICF patients have a much shorter mean telomere length than their parental controls. ICF patients also have a higher frequency of chromosome ends with telomeric signal loss and DNA damage foci. As expected, primary cells from ICF patients undergo accelerated telomere shortening in culture, leading to premature replicative senescence with abnormally low population doublings [141,142].

Given the significantly increased levels of TERRAs, it is speculated that these phenotypes are linked to TERRA R-loop-mediated DNA damage and loss of HR repair. Indeed, ICF cells have more R-loop than wild-type cells, confirming that higher TERRA levels result in increased R-loop formation at telomeres [142]. Using telomere FISH combined with spectral karyotyping, Selig’s group characterized telomere length shortening at each chromosome end. They found that telomere lengths in ICF cells are abnormally heterogeneous, with twelve telomeres significantly shorter than control groups and nine telomeres similar to or even longer than control groups. Intriguingly, they showed that not all telomere ends have the same risk of length shortening. A subset of telomeres (six telomere ends) is susceptible to shortening [143]. Whether this non-random feature of telomere length distribution correlates with individual subtelomeric CpG density, GC-skewness, or other chromatin modification is worth future investigation.

### 4.4. Connection of Oxidative DNA Damage and R-Loop Accumulation

Telomeres are well known for posing a challenge to replication machinery due to their long repetitive sequences and secondary structure-prone nature. At the same time, the high guanine content of telomere sequences makes them a preferred target for oxidative damage. In fact, oxidative stress is thought to be one of the most common mechanisms underlying telomere shortening [144,145]. While numerous cues are associated with increased cellular reactive oxygen species (ROS), inflammation and mitochondrial impairment are the two primary sources of ROS in cells [146,147]. Previous studies from other groups and ours have demonstrated that patients with dyskeratosis congenita (DC) and murine models with critically short telomeres exhibit impaired mitochondrial function and elevated ROS levels [148,149,150].

ROS can induce direct oxidized bases, single strand breaks (SSB), and double strand breaks (DSB) at telomeres [144]. If not properly repaired, the lesions will give rise to telomere fragility and/or telomere loss [145,151,152,153]. However, little is known about how oxidative DNA lesions affect R-loops at telomeres. Lan’s group utilized a light-sensitive fusion protein of KillerRed (KR) and TRF1 (KR-TRF1) to introduce oxidative DNA damage locally at telomeres. They discovered that ROS-induced DNA strand breaks (SSBs and/or DSBs) contribute to the accumulation of R-loops in a TERRA- and TRF2-dependent manner. However, when the Fok1-TRF1 fusion protein was used, which is known to induce DSBs at telomeres, the induction of R-loop formation was much less efficient, indicating that SSBs induced by ROS play a significant role in stimulating R-loop formation. R-loops are then recognized by a DNA repair protein, CSB, which promotes subsequent recruitment of RAD52. Together with POLD3, this ROS-induced DNA damage is eventually repaired by a CSB-RAD52-POLD3-mediated break-induced DNA replication (BIR) pathway [154].

## 5. Conclusions

The general R-loop structure was discovered over four decades ago and has since been shown to play a vital role in various cellular functions. The finding that TERRA RNA can invade telomeric dsDNA and form telomeric R-loop structures provides more insights to our understanding of RNA-dependent metabolism, including transcription, processing, and repair. For instance, unlike the conventional view of co-transcriptional-mediated R-loop formation in cis, TERRA-mediated R-loop formation can occur post-transcriptionally in trans. Over the last decade, it has become clear that TERRA R-loops play an important role in telomere length regulation and end protection.

One of the most important characteristics of telomeric R-loops is their dynamic maintenance. A finely tuned R-loop level is essential to telomere integrity. The transient formation of R-loops in ALT cancer cells provokes replication stress, but it also signals an early stage of DNA repair by recruiting RAD51, BRCA1, and other repair factors and initiating HR-mediated telomere synthesis induced by DNA strand breaks. To complete HR-mediated telomere synthesis, the existing R loops must be efficiently removed. Thus, ALT survival requires a dynamic switch between R-loop formation and resolution at telomeres. In non-ALT cancer cells, telomeric R-loop levels are more correlated with cell cycle regulation, allowing telomere-associated R-loops to be resolved in time to avoid transcription and replication collision risk. Conversely, loss of dynamic regulation results in the accumulation of persistent or unscheduled R-loops at telomeres, which becomes one of the major drivers of genome and telomere instability, as seen in yeast cells and human ICF patient cells.

In conclusion, the balanced formation and resolution of telomeric R-loops hold a key area of future research due to their close association with human diseases and the potential for developing R-loop-based therapeutics to target ALT cancers.
